# PAD-UFES-20: A skin lesion dataset composed of patient data and clinical images collected from smartphones

**DOI:** 10.1016/j.dib.2020.106221

**Published:** 2020-08-25

**Authors:** Andre G.C. Pacheco, Gustavo R. Lima, Amanda S. Salomão, Breno Krohling, Igor P. Biral, Gabriel G. de Angelo, Fábio C.R. Alves Jr, José G.M. Esgario, Alana C. Simora, Pedro B.C. Castro, Felipe B. Rodrigues, Patricia H.L. Frasson, Renato A. Krohling, Helder Knidel, Maria C.S. Santos, Rachel B. do Espírito Santo, Telma L.S.G. Macedo, Tania R.P. Canuto, Luíz F.S. de Barros

**Affiliations:** a Graduate Program in Computer Science, Federal University of Espírito Santo, Vitória, Brazil; b Nature Inspired Computing Laboratory, Federal University of Espírito Santo, Vitória, Brazil; c Dermatological and Surgical Assistance Program (PAD), Federal University of Espírito Santo, Vitória, Brazil; d Faculty of Medicine, Federal University of Espírito Santo, Vitória, Brazil; e Department of Specialized Medicine, Federal University of Espírito Santo, Vitória, Brazil; f Production Engineering Department, Federal University of Espírito Santo, Vitória, Brazil; g Secretary of Health of the Espírito Santo state, Governor of Espírito Santo state, Vitória, Brazil; h Pathological Anatomy Unit of the University Hospital Cassiano Antônio Moraes (HUCAM), Federal University of Espírito Santo, Vitória, Brazil

**Keywords:** Skin cancer, Skin lesion, Clinical data, Cancer research, Computer-Aided Diagnosis (CAD)

## Abstract

Over the past few years, different Computer-Aided Diagnosis (CAD) systems have been proposed to tackle skin lesion analysis. Most of these systems work only for dermoscopy images since there is a strong lack of public clinical images archive available to evaluate the aforementioned CAD systems. To fill this gap, we release a skin lesion benchmark composed of clinical images collected from smartphone devices and a set of patient clinical data containing up to 21 features. The dataset consists of 1373 patients, 1641 skin lesions, and 2298 images for six different diagnostics: three skin diseases and three skin cancers. In total, 58.4% of the skin lesions are biopsy-proven, including 100% of the skin cancers. By releasing this benchmark, we aim to support future research and the development of new tools to assist clinicians to detect skin cancer.

## Specifications Table

**Table:** 

Subject	Cancer Research and Computer Vision and Pattern Recognition
Specific subject area	Automated Skin Cancer detection
Type of data	Images and Metadata
How data were acquired	All data were collected through smartphone devices using an application developed specifically to this work
Data format	Portable Network Graphics (PNG) and Comma Separated Values (CSV) file formats
Parameters for data collection	All data are collected during the patient appointment
Description of data collection	Each sample in this dataset consists of an clinical image and a set of patient clinical data that contains up to 21 features
Data source location	Institution: Federal University of Espírito Santo (UFES) Region: Espírito Santo Country: Brazil
Data accessibility	Dataset is available on https://data.mendeley.com/datasets/zr7vgbcyr2/1
Related research article	Andre G. C. Pacheco and Renato A. Krohling, ”The impact of patient clinical information on automated skin cancer detection.” Computers in biology and medicine 116 (2020): 103545. https://doi.org/10.1016/j.compbiomed.2019.103545

## Value of the Data

• This dataset is useful to support future research and the development of new tools to detect skin cancer without using dermoscopy images.
• Automated skin cancer detection using dermoscopy images is an important and promising task. However, in emerging countries and in remote/rural areas there is a strong lack of medical tools and experts to assist the population. Thus, this dataset is an effort to assist researchers to develop tools, in particular, to assist skin cancer detection in these types of areas.
• This dataset may be used as a training data to develop Computer-Aided Diagnosis (CAD) systems to deal with skin cancer.
• Also, this data may be helpful for educational purposes, i.e., to train medical students to identify skin cancer.
• Beyond the clinical images collected from smartphones, this dataset also contains the patient clinical data related to each image. It may help researchers to understand the relationship between these two types of data and how they can be used to improve skin cancer detection.

## 1 Data

### 1.1 Overview

Over the past few years, different skin lesion datasets composed of dermoscopy images have been fomenting the development of CAD systems for skin cancer analysis [1]. The Atlas of Dermoscopy [2] was the first well-known dataset containing over one thousand skin lesion images. In 2018, Tschandl, Rosendahl, and Kittler [3] released the HAM10000 dataset, a large collection of multi-source dermoscopy images of common pigmented skin lesions containing over 10 thousand samples. One year later, Combalia et al. [4] presented the BCN20000 dataset, which contains around twenty-thousand images of skin cancer, including lesions found in hard-to-diagnose locations (nails and mucosa). Together, both HAM10000 and BCN20000 made up the majority part of the International Skin Imaging Collaboration (ISIC) archive^1^, a public repository that plays an important role for both the purposes of clinical training and for supporting technical research toward automated algorithmic analysis. Since 2016, this organization hosts the ISIC challenge, an open competition that has been boosting automated skin cancer detection algorithms. Recently, the ISIC 2020 challenge^2^ was released using 33,126 dermoscopy training images of unique benign and malignant skin lesions from over 2000 patients from the archive.

Developing CAD systems to detect skin cancer using dermoscopy images is an important and promising task [5]. However, in emerging countries [6] and in remote/rural areas [7] there is a strong lack of medical tools and experts to assess such type of data. In these places, CAD systems embedded on smartphones may be a low-cost solution, in particular, to assist non-expert clinicians to detect skin cancer [8]. Alves et al. [9] attached a special type of dermatoscope to smartphones to collect a skin lesion dataset using different levels of focus. However, this dataset is not publicly available nor annotated by experts, and it still depends on the dermatoscope, which is costly and may not be available in emerging countries. Side-stepping the dependence on dermatoscope, Udrea et al. [10] used the SkinVision^3^ database to gather a large skin lesion dataset collected from smartphone cameras (clinical images). However, this is a private dataset. In addition, the skin lesion diagnostic is obtained only from the raw images, there are no biopsy-proven samples, and the dataset does not contain patient demographics.

In order to support future research and the development of new tools to detect skin cancer, we present the PAD-UFES-20, a dataset composed of clinical images of skin lesions and patient clinical data related to each skin lesion collected from different smartphone devices. The dataset contains 2298 samples of six different types of skin lesions, three cancers and three skin diseases. In addition, each image has up to 21 patient clinical features including the patient’s age, skin lesion location, Fitzpatrick skin type, and skin lesion diameter. The dataset was collected from 2018 to 2019 and, to the best of our knowledge, is the first skin lesion archive that: (1) it is publicly available, (2) contains clinical images collected from smartphones, (3) includes patient demographics.

### 1.2 Description

All samples within PAD-UFES-20 represents a skin lesion of a patient that is composed of an image and a set of metadata. A patient may have one or more skin lesions and a skin lesion may have one or more images. In total, there are 1373 patients, 1641 skin lesions, and 2298 images present in the dataset. Although the number of total samples is not as high as the HAM10000 or BCN20000 datasets, we would like to highlight that this is the first step towards building a public dataset of this type. For comparative purposes, the ISIC archive started in 2016 [11] containing only 1279 samples, and today it is over 40 thousand samples. Moreover, we aim to update the dataset every two years by including more samples and more skin lesions. All data records are publicly available at Mendeley Data [12].

#### 1.2.1 Images

Since the images in this dataset are collected using different smartphone devices they present different resolutions, sizes, and lighting conditions. Essentially, an application to detect skin cancer using clinical images needs to deal with such variability. Thus, it aims to simulate the real world. All images are available in Portable Network Graphics (PNG) file format. In addition, all images are raw, i.e., we do not apply any image processing to enhance visualization.

#### 1.2.2 Meta-data

The metadata associated with each skin lesion is composed of 26 attributes: 21 patient clinical features, four identifying features (patient ID, lesion ID, Image ID, and if the sample is biopsy-proven), and a diagnostic label. All attributes are available in a Comma Separated Values (CSV) file format, where each line represents a skin lesion and each column a metadata attribute. We describe all attributes in Table 1.

**Table 1:** Description of each attribute present in the metadata CSV file.

Attribute	Description
patient_id	a string representing the patient ID – example: PAT_1234
lesion_id	a string representing the lesion ID – example: 123
img_id	a string representing the image ID, which is a composition of the patient ID, lesion ID, and a random number – example: PAT_1234_123_000
smoke	a boolean to map if the patient smokes cigarettes
drink	a boolean to map if the patient consumes alcoholic beverages
background_father and background_mother	a string representing the country in which the patient’s father and mother descends. Note: many patients descend from Pomerania, a region between Poland and Germany. Although it is not a country, we decided to keep the nomenclature, since they identify themselves as Pomeranians descendants.
age	an integer representing the patient’s age
pesticide	a boolean to map if the patient uses pesticides
gender	a string representing the patient’s gender
skin_cancer_history	a boolean to map if the patient or someone in their family has had skin cancer in the past
cancer_history	a boolean to map if the patient or someone in their family has had any type of cancer in the past
has_piped_water	a boolean to map if the patient has access to piped water in their home
has_sewage_system	a boolean to map if the patient has access to a sewage system in their home
fitspatrick	a integer representing the Fitspatrick skin type [13]
region	a string representing one of the 15 macro-regions previously described
diameter_1 and diameter_2	a float representing the skin lesions’ horizontal and vertical diameters
diagnostic	a string representing the skin lesion diagnostic – BCC, SCC, ACK, SEK, MEL, or NEV
itch	a boolean to map if the skin lesion itches
grew	a boolean to map if the skin lesion has recently grown
hurt	a boolean to map if the skin lesion hurts
changed	a boolean to map if the skin lesion has recently changed
bleed	a boolean to map if the skin lesion has bled
elevation	a boolean to map if the skin lesion has an elevation
biopsed	a boolean to map if the diagnostic comes from clinical consensus or biopsy

It is important to note that some attributes may be missing for some skin lesions. In brief, patient_id, lesion_id, img_id, age, region, and biopsed are always present. The remaining attributes depend on the patient’s answers during the appointment. Missing values are left blank in the CSV file. When a patient does not know the answer for some question – for example, he/she does not know their father’s background – we fill the feature as UNK (unknown).

## 2 Materials and methods

The Dermatological and Surgical Assistance Program (in Portuguese: Programa de AssistȬncia Dermatolgica e Cirurgica - PAD) at the Federal University of Espírito Santo (UFES) is a nonprofit program that provides free skin lesion treatment, in particular, to low-income people who cannot afford private treatment. For historical reasons, the Espírito Santo state has received thousands of immigrants from Europe throughout the 19th century. As Brazil is a tropical country, most of these immigrants and their descendants were/are not adapted to this climate. As a result, there is a high incidence of skin lesions/cancer in this state and the PAD plays a fundamental role to assist these people [14].

In late 2017, the Nature Inspired Computing Laboratory (LABCIN-UFES) and the PAD started a partnership that resulted in the creation of a web-based platform and a multi-platform smartphone application to collect and store patient clinical data and skin lesion images. In this section, we present a brief description of this system and the data collection.

### 2.1 Software description

**Fig. 1:**
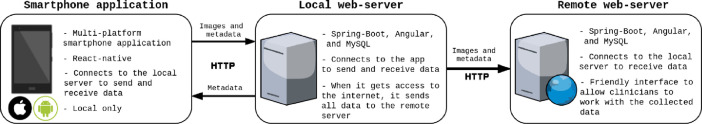
An illustration of the software structure that we developed to collect data at the PAD.

The PAD provides full skin lesion treatment, from the screening to the surgical process (if needed), in 11 different countryside cities in Espírito Santo state. Most of these places are rural areas without Internet access, which is an important requisite to take into account. In this context, the software infrastructure is composed of three parts: a local web-server, a remote web-server, and a multi-platform smartphone application. Basically, all data is collected using the smartphone application, which locally connects to the local web-server to store data. After the data collection is done, as soon as the local web-server gets access to the internet, it synchronizes all data with the remote one. This software structure is illustrated in Fig. 1.

Regarding technologies applied to develop the software, the smartphone application was developed using React-Native^4^, which is an open-source library, based on Javascript^5^, for building user interfaces for both Android and iOS. Both local and remote web-servers were developed using two main frameworks: Angular^6^ and Spring-Boot^7^. Angular is a open-source framework, based on Typescript^8^, for developing efficient and sophisticated single-page applications. Spring-Boot is also an open-source framework, based on Java^9^, that is used to create a micro service on the server side. The database is managed using MySQL^10^.

Beyond storing all data in an organized and structured way, the remote web-server offers a friendly interface to clinicians to access the collected data. This is important for three reasons: (1) it is used to train medical students to identify the lesions; (2) it is important to keep tracking patient lesions since evolution is an important feature to pay attention to detect skin cancer [15]; (3) it helps clinicians with statistics about skin lesions and patients, which is relevant to understand the behavior of the disease over Espírito Santo state. This software is also open-sourced and the code is publicly available on Github^11^.

### 2.2 Data collection

**Fig. 2:**
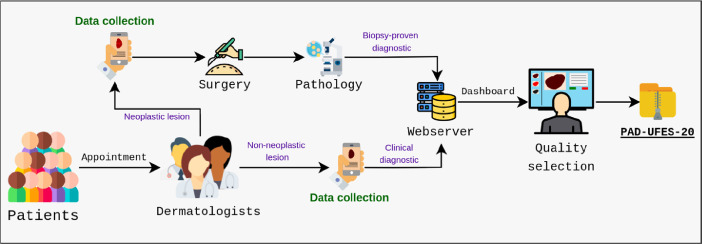
Data collection workflow of the PAD-UFES-20 dataset.

Fig. 2 summarizes our data collection workflow. First of all, the patients have an appointment with a group of up to three senior dermatologists (at least 15 years of experience) that assesses the skin lesion. If the group identifies a neoplasm, the skin lesion is removed through surgical procedure – performed by medical students under the supervision of two senior plastic surgeons of the PAD – and sent to the Pathological Anatomy Unit of the University Hospital Cassiano Antȳnio Moraes (HUCAM) at the UFES to perform histopathology examination. On the other hand, if the group has a consensus that there is no neoplasm, they do not request a biopsy. In both cases, we collect images and clinical data. Later, when the biopsy result is available, it is filled for those lesions in which it was requested. All data is stored in a web-server and the final step is a quality selection to review every single sample that was collected in the previous steps.

The goal of the quality selection step is to review the patient clinical data and remove poor quality images. All data from the appointment are filled by senior medical students and the images are collected using different types of smartphone devices. In addition, the smartphone application allows the user to crop the image in order to select only the region of interest. As a result, images in different conditions are upload to the server. Thereby, during the quality selection, we delete those images according to the following rules:

• The image resolution is very poor and it is not possible to identify the lesion
• The patient may be identified because of a tattoo, for example
• The lesion is completely occluded by hair or ink marking

Regarding the patient clinical data, we review all samples to correct typos and information that are clearly wrong, for example, birth dates before 1900 or skin lesions’ diameter that are much bigger than it looks on the image according to visual inspection. For these cases, we re-checked the physical files in order to fix the information. If it is not possible to fix it, we remove the wrong information from the clinical data and it becomes missing data. Lastly, we translate all clinical data from Brazilian-Portuguese to English.

### 2.3 Data selection

**Fig. 3:**
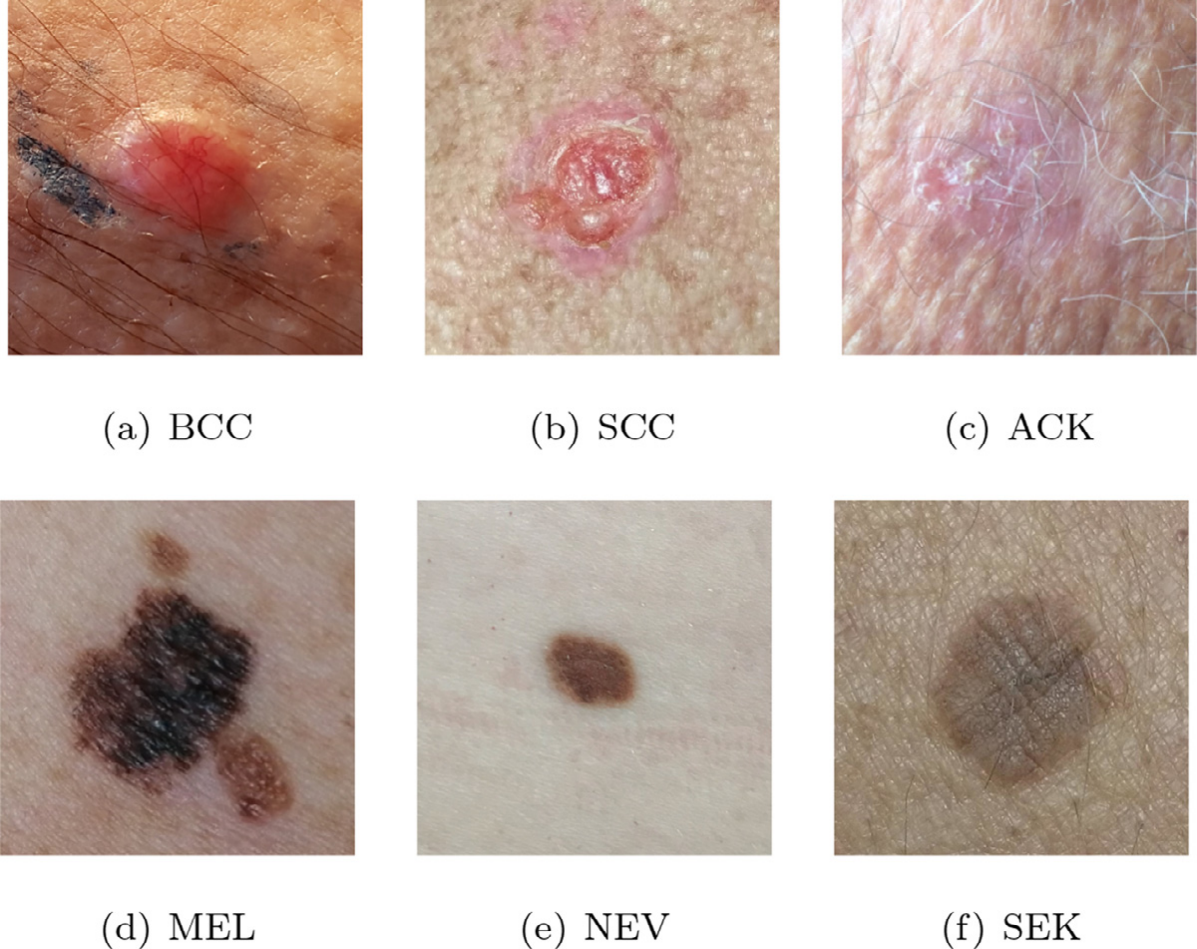
A sample of each type of skin lesion present in PAD-UFES-20 dataset.

The dataset was collected during 2018 and 2019. In total, there are over 50 types of skin lesions that were collected during this period. However, most of them are rare and contain only a few samples. For this reason, we selected the seven most common skin lesions diagnosed at PAD, which are: (1) Basal Cell Carcinoma (BCC), (2) Squamous Cell Carcinoma (SCC), (3) Actinic Keratosis (ACK), (4) Seborrheic Keratosis (SEK), (5) Bowens disease (BOD), (6) Melanoma (MEL), and (7 Nevus (NEV). As the Bowens disease is considered SCC in situ [13], we clustered them together, which results in six skin lesions in the dataset, three skin cancers (BCC, MEL, and SCC) and three skin disease (ACK, NEV, and SEK). An image sample and the quantity of each of these six skin lesions present in PAD-UFES-20 dataset are presented in Fig. 3 and in Table 2, respectively. It is important to note that all samples diagnosed as skin cancer are proved by biopsy. For those cases in which the pathology yielded a biopsy that is inconclusive, we removed the sample from the dataset. In Table 2 is also described the percentage of samples diagnosed as ACK, NEV, and SEK that are also proved by biopsy. As presented in Table 1, we provide this information in the metadata file through the biopsy attribute.

**Table 2:** The number of samples and the % of biopsy-proven for each type of skin lesion present in PAD-UFES-20 dataset.

Diagnostic	***N*****æ of samples**	% biopsied
Actinic Keratosis (ACK)	730	24.4%
Basal Cell Carcinoma of skin (BCC)	845	100%
Malignant Melanoma (MEL)	52	100%
Melanocytic Nevus of Skin (NEV)	244	24.6%
Squamous Cell Carcinoma (SCC)	192	100%
Seborrheic Keratosis (SEK)	235	6.4%
**Total**	**2298**	**58.4%**

To conclude, there are approximately 120 different anatomical regions used by the PAD’s dermatologists and pathologists. We clustered these regions in 15 macro-regions that are more frequent and have more potential to raise a skin lesion, such as follows: face, scalp, nose, lips, ears, neck, chest, abdomen, back, arm, forearm, hand, thigh, shin, and foot. As skin lesions have preferences for some regions of the body [13], [15], it is an important feature to consider.

### 2.4 Technical validation

The histopathology procedure involves the following steps: (1) the collection of a skin fragment, (2) tissue fixation in formaldehyde (at a concentration of 10%), (3) macroscopic analysis of the skin fragment, (4) histological processing, (5) producing the microscope slides, and (6) a microscopic study with diagnosis’ formulation and interpretation [16]. As described in Table 2, 58.4% of the skin lesions in PAD-UFES-20 dataset are biopsy-proven. This number is compatible with other skin lesion datasets described in literature, for instance, the HAM10000 dataset [3] has 53.3% of biopsy-proven samples.

Regarding the metadata features, they were collected according to the anamnesis of a patient, which beyond the skin lesion screening, dermatologists also consider the anatomical region, diameter, ulceration, itching, bleeding, among others characteristics of the skin lesion [13], [15]. In addition, risk factors are also taken into accounts such as exposure to chemicals, cancer history, and the type of the skin [17]. Combining patient clinical data and skin lesion images has been shown to improve the efficacy of CAD systems for skin cancer detection [5], [18].

### 2.5 Additional notes

As we previously stated, there are two main characteristics that differ the PAD-UFES-20 dataset from other skin lesion datasets available on literature: the clinical images collected from smartphone devices and the set of patient clinical data. Beyond educational purposes, this dataset aims to support the development of CAD systems embedded in smartphones, in particular, to assist clinicians/non-experts to handle skin lesions in remote places. In addition, the dataset contains samples of six of the most commonly known skin lesions [13], including pigmented and non-pigmented ones.

**Fig. 4:**
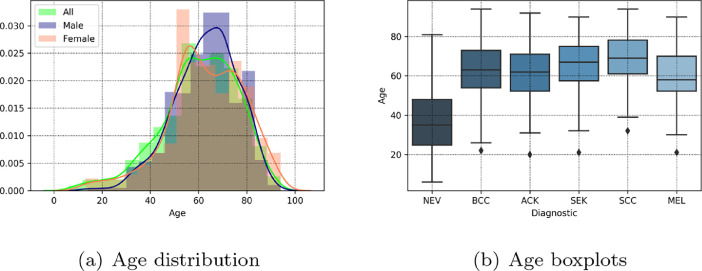
The patients age distribution according to gender and the age boxplots for each diagnostic.

All data are collected in 11 different cities in Espírito Santo state, Brazil. Most of the patients are European immigrants descendants and are or have been farm workers with many hours of sun exposure per day. In addition, the patient average age is approximately 60 years old, but it may vary according to the diagnostic – see Fig. 4. Thus, it is important to note this data represents a specific population in a particular region in Brazil.

Another important aspect is the raw image. We do not apply any image processing algorithm into the collected images. A suggestion to enhance the images would be using color constancy algorithms, which have been shown to be helpful for automated skin cancer detection [5], [19]. Also, as we may note in Table 2, the dataset is imbalanced, in particular, for melanoma, the deadliest case of skin cancer. Unfortunately, imbalanced datasets are quite common for skin lesion datasets. For instance, in HAM10000 [3] and BCN20000 [4] datasets, we may find an imbalance among the diagnostic labels of approximately 58:1 and 40:1, respectively. For automated skin cancer detection, common algorithms to deal with this issue are oversampling and weighted loss functions [5].

Lastly, in supplementary materials, we provide the medical questionnaire that results in the patient’s clinical data and a CSV file in which we link all medical terms used in this paper with the SNOMED CT concepts^12^.

## Ethics statement

The dataset was collected along with the Dermatological and Surgical Assistance Program (PAD) of the Federal University of Espírito Santo. The program is managed by the Department of Specialized Medicine and was approved by the university ethics committee (næ 500002/478) and the Brazilian government through Plataforma Brasil (næ 4.007.097), the Brazilian agency responsible for research involving human beings. In addition, all data is collected under patient consent and the patients privacy is completely preserved.

## Declaration of Competing Interest

The authors declare that they have no known competing financial interests or personal relationships which have, or could be perceived to have, influenced the work reported in this article.

## Appendix A Supplementary materials

Supplementary Data S1 Supplementary Raw Research Data. This is open data under the CC BY license http://creativecommons.org/licenses/by/4.0/ Supplementary Data S1

Supplementary Data S2 Supplementary Raw Research Data. This is open data under the CC BY license http://creativecommons.org/licenses/by/4.0/ Supplementary Data S2

Supplementary Data S3 Supplementary Raw Research Data. This is open data under the CC BY license http://creativecommons.org/licenses/by/4.0/ Supplementary Data S3

## Footnotes

1 https://www.isic-archive.com

2 https://challenge2020.isic-archive.com

3 https://www.skinvision.com/

4 https://reactnative.dev

5 https://www.javascript.com

6 https://angular.io

7 https://spring.io

8 https://www.typescriptlang.org

9 https://www.java.com

10 https://www.mysql.com

11 https://github.com/labcin-ufes/PAD-UFES-20

12 https://www.snomed.org/
