# Supplementary material for: PAD-UFES-20: A skin lesion dataset composed of patient data and clinical images collected from smartphones
Source: Data Brief. 2020 Aug 25;32:106221. doi: 10.1016/j.dib.2020.106221 (PMC7479321; doi:10.1016/j.dib.2020.106221)
Supplement: Supplementary Data S2 — Supplementary Raw Research Data. This is open data under the CC BY license http://creativecommons.org/licenses/by/4.0/ [file mmc2.pdf]

FEDERAL UNIVERSITY OF ESPÍRITO SANTO

DERMATOLOGICAL AND SURGICAL ASSISTANCE PROGRAM

CLINICAL APPOINTMENT QUESTIONNAIRE

Patient´s full name:____

Healthcare ID Number: ____

SESSION 1 – QUESTIONS ABOUT THE PATIENT

1) Age: ______ years.

2) Gender: ( ) Male ( ) Female

3)Father's descendancy: ____

4) Mother’s descendancy: ____

5) Does your house have piped water? ( ) Yes ( ) No

6) Does your house have a sewer system? ( ) Yes ( ) No

7) Do you smoke? ( ) Yes ( ) No

8) Do you drink alcohol? ( ) Yes ( ) No

9) Have you ever had skin cancer? ( ) Yes ( ) No

10) Has anyone in your family ever had any type of cancer? ( ) Yes ( ) No

11) Do you use pesticides at work? ( ) Yes ( ) No

SESSION 2 – QUESTIONS ABOUT THE SKIN LESION

1) Fitzpatrick Scale: ( ) I ( ) II ( ) III ( ) IV ( ) V ( ) VI

2) Anatomical region: ____

3) Horizontal diameter: ____

4) Vertical diameter: ____

5) Has it itched? ( ) Yes ( ) No

6) Has it grown? ( ) Yes ( ) No

7) Does it hurt? ( ) Yes ( ) No

8) Has it changed its appearance? ( ) Yes ( ) No

9) Has it bled? ( ) Yes ( ) No

10) Does it have an elevation? ( ) Yes ( ) No

11) Clinical diagnostic:

____

____

SESSION 3 – ANATOMOPATHOLOGICAL EXAMINATION*

Result of excisional biopsy:

____

____

____

____

* If applicable.

Date: ____.

STAMP AND SIGNATURE OF THE PHYSICIAN
